# Valuing Australian parent preferences for community-based nutrition and physical activity initiatives: a discrete choice experiment

**DOI:** 10.1093/heapro/daag033

**Published:** 2026-03-09

**Authors:** Nicole Ward, Thao Thai, Melanie Nichols, Marj Moodie, Kim Robinson, Vicki Brown

**Affiliations:** Deakin Health Economics, Institute for Health Transformation, Deakin University, 1 Gheringhap St, Geelong, Victoria 3220, Australia; Global Centre for Preventive Health and Nutrition, Institute for Health Transformation, Deakin University, 1 Gheringhap St, Geelong, Victoria 3220, Australia; Health Economics Group, School of Public Health and Preventative Medicine, Monash University, 900 Dandenong Road, Melbourne, Victoria 3145, Australia; Global Centre for Preventive Health and Nutrition, Institute for Health Transformation, Deakin University, 1 Gheringhap St, Geelong, Victoria 3220, Australia; Deakin Health Economics, Institute for Health Transformation, Deakin University, 1 Gheringhap St, Geelong, Victoria 3220, Australia; Global Centre for Preventive Health and Nutrition, Institute for Health Transformation, Deakin University, 1 Gheringhap St, Geelong, Victoria 3220, Australia; School of Health and Social Development, Deakin University, 1 Gheringhap St, Geelong, Victoria 3220, Australia; Deakin Health Economics, Institute for Health Transformation, Deakin University, 1 Gheringhap St, Geelong, Victoria 3220, Australia; Global Centre for Preventive Health and Nutrition, Institute for Health Transformation, Deakin University, 1 Gheringhap St, Geelong, Victoria 3220, Australia

**Keywords:** discrete choice experiment, obesity, climate change, community

## Abstract

Parent engagement in community-based obesity prevention interventions (CBOPIs) enhances obesity prevention outcomes. Actions from CBOPIs may also have climate change-related impacts, but little is known about specific elements of CBOPIs that promote parental engagement, and whether parents prefer CBOPIs that aim to address obesity alone or obesity and climate change warrants further investigation. An unlabelled 12-choice task discrete choice experiment (DCE) was undertaken, using a D-efficient design and incorporating two CBOPI alternatives plus an opt-out alternative. CBOPIs were described by six attributes: cost, aim, involvement, effectiveness, convenience, and social opportunities. Attributes were informed by a literature review of enablers and barriers to parent participation. The survey was electronically distributed to parents of primary-school-aged children in each Australian state and territory during April–May 2024. Data were analysed using conditional logit models, and willingness-to-pay for attributes was estimated. Parents (*n* = 438) preferred less costly CBOPIs (*P* < .001) that aim to address both healthy lifestyles and climate change (*P* = .086). Parents preferred short-term, manageable disruptions to family schedules to accommodate CBOPI participation (*P* = <.001) over no intervention. They were willing to pay an additional $3.97 per fortnight (∼$104.000 per year) for CBOPI participation to be convenient (standard error 1.380, *P* = .004). Our findings suggest that parents preferred CBOPIs that aim to address both healthy lifestyles and climate change. This suggests that incorporating climate change action into CBOPIs may increase parent support and consequently support CBOPI outcomes for both obesity prevention and climate change action.

Contribution to Health Promotion

- Obesity prevention and climate change action are significant public health issues that may be addressed with community-based obesity prevention interventions
- Australian parents support climate change action as part of community-based obesity prevention interventions
- These findings support the inclusion of climate change in community-based obesity prevention interventions, and this may assist design interventions that increase parent support

## Background

Community-based obesity prevention interventions (CBOPIs), particularly those involving primary school aged children, have been successful in reducing obesity (Bleich *et al*. 2013). CBOPIs focus on improving diets and/or increasing physical activity levels in specific communities through positive and supportive changes across multiple settings. CBOPIs are usually evaluated using physical health measures (e.g. changes in body weight (Sultana *et al*. 2023)). Intervention content and messaging commonly focus on healthy lifestyles rather than obesity prevention, to foster positive engagement (Zeb *et al*. 2024), while recognizing that lifestyle behaviours themselves are shaped by broader structural and systemic determinants(Sultana *et al*. 2024)

CBOPIs include actions that may also influence another important public health issue—climate change (Ward *et al*. 2025). Obesity and climate change are two of the most significant global health issues (Swinburn *et al*. 2019); however, recognition of the bidirectional relationship between obesity prevention and climate change actions is a relatively recent development in the literature and may not be widely acknowledged by children or their parents. Actions that influence both obesity and climate change are known as double-duty actions (Swinburn *et al*. 2019). Whilst double-duty actions have been identified at the macro level, there is limited evidence at the community level, and it is uncertain if actions for obesity prevention may yield climate change impacts. An example of a double-duty action is increasing active transport, where potential health benefits are gained from increased physical activity, along with potential environmental benefits from reduced vehicle emissions (Ward *et al*. 2025). Other examples of double-duty actions in CBOPIs include CBOPIs that increase locally produced fruit and vegetables, increase green space, and reduce energy in food production (Ward *et al*. 2025).

Double-duty actions may be already occurring in CBOPIs but without being explicitly recognized for their double-duty benefits (Ward *et al*. 2025). The Whole of Systems Trial of Prevention Strategies for Childhood Obesity (WHO STOPS) was a 4-year CBOPI involving 10 communities in regional Victoria, Australia. WHO STOPS aimed to reduce childhood obesity through a systems approach that facilitated community engagement (Allender *et al*. 2016). WHO STOPS did not explicitly include any climate-focused objectives; however, several of the strategies were double-duty actions (Ward *et al*. 2025). The climate change benefits were not recognized by the study but were identified through a separate study published in 2024 (Ward *et al*. 2025). This study developed a framework to identify double-duty actions in CBOPIs, and it provides a comprehensive overview of double-duty actions and how CBOPIs may have climate change benefits in addition to health benefits. Notably, whilst the impact of individual CBOPIs on climate change mitigation is likely minimal, the recognition of climate change benefits is important to build and promote collective action on climate change. Further research is required to quantify potential emissions from CBOPIs. However, climate change is a significant contemporary public health issue, which may potentially influence stakeholder engagement as climate change benefits are supported by parents particularly when their child is motivated to change behaviour due to climate change (Fornwagner and Hauser 2022).

Interventions that attract parent and caregiver participation and support are likely to have more successful outcomes (Yavuz *et al*. 2015, Bryant *et al*. 2017). Throughout this paper, the term ‘parents’ is used to refer collectively to both parents and nonparental primary caregivers. Parents influence the actions, behaviours, and environments of primary-school-aged children. Family engagement is essential for the acceptability and success of CBOPIs, in part by enabling CBOPI messaging to be reinforced at home (Clarke *et al*. 2015). However, Smith *et al.* (2014) found commitment to making healthy lifestyle changes, and intervention participation was challenging for parents/participants. Currently, there is limited literature on which factors enable and motivate parents to engage in CBOPIs to support both their child’s participation and intervention success (Jurkowski *et al*. 2013). Specifically, it is unknown if parents and caregivers would be more willing to support an initiative that focuses solely on healthy lifestyles (healthy diet and active living through nutrition and physical activity strategies), or more willing to support an initiative that explicitly focuses on both climate change and healthy lifestyle objectives.

The aim of this research is to identify influences on parent support for CBOPIs using a discrete choice experiment (DCE), and to determine which intervention focus (healthy lifestyle or healthy lifestyle and climate change) parents prefer. To our knowledge, there are no published DCEs on parent preferences for attributes describing CBOPIs. This evidence is needed to inform how CBOPIs should be presented to parents to generate the most interest and support, and to direct the inclusion of climate change action in CBOPIs.

## Methods

This study was approved by the Deakin University Human Research Ethics Advisory Group (HEAG-H 11_2024). Best practice guidelines were followed for the DCE design, conduct, and analysis (Hauber *et al*. 2016, Ride *et al*. 2024).

A DCE measures participant preferences based on the theory that participants make choices that maximize their utility (or provide the greatest benefits) (Lancsar *et al*. 2017). DCEs are a type of stated preference method used to elicit individuals’ preferences by presenting them with a series of hypothetical scenarios. In each scenario, participants are asked to choose their preferred option from a set of alternatives. Each alternative is described by a combination of attributes relevant to the decision-making context. In this study, attributes are the factors that pertain to parent support for CBOPIs. Each attribute varies across a set of levels, representing the specific values or categories the attribute can take. By systematically varying the attribute levels across choice sets, DCEs allow researchers to assess the relative importance of different attributes and the trade-offs individuals are willing to make between them (Lancsar *et al*. 2022).

### Identification and development of DCE attributes and levels

The DCE attributes were identified through a literature search on barriers and enablers to parent and caregiver participation in CBOPIs and the study aims. Four databases (Academic Search Complete, MEDLINE Complete, CINAHL Complete and Global Health) were searched in September 2023 using subject terms ‘obesity prevention intervention’ AND ‘community-based participation OR engagement OR involvement’ AND ‘parents OR caregivers OR mother OR father OR parent’. Included studies were full-text articles published in English between 2018 and 2023 to ensure contemporary influences were identified. Studies were excluded if they presented a treatment or diagnostic approach, or an intervention for children outside the 4- to 12-year age bracket.

Seventeen enablers and four barriers were initially identified from seven studies (Davison *et al*. 2013, Jurkowski *et al*. 2013, Clarke *et al*. 2015, St George *et al*. 2018, Waters *et al*. 2018, Brown *et al*. 2019, White *et al*. 2019). The identified barriers and enablers from the literature search were extracted into a Microsoft Excel spreadsheet. Barriers and enablers were then grouped into like terms; for example, ‘cost’ and ‘financial constraints’ were grouped into ‘cost’.

Barriers and enablers that could be potentially modified by those developing and implementing CBOPIs were reviewed for inclusion by N.W. and V.B. (Supplementary file 1). For example, time and location of initiative was shortlisted, whilst lack of space at home was not.

To minimize the cognitive burden for participants, the attributes and levels were refined through researcher expert opinion and pretesting through individual interviews with a convenience sample of parents (*n* = 13), to determine comprehension, clarity, and prioritization. This ensured attributes and levels were consistently interpreted and realistic (Bridges *et al*. 2011).

The final DCE had six attributes. This was slightly above the median number of five attributes that is common in health DCEs (Soekhai *et al*. 2019). Four attributes (cost, involvement, effectiveness, and convenience) were identified from the literature search and a further two from the research question (aim) and pilot of the study with parents (social). Attributes were tested with parents. Parents (*n* = 4) were asked to rank the six attributes in order of importance to confirm that all included attributes were relevant to them. Any attribute that was consistently ranked last (i.e. least important) by all parents would have been removed from the survey as it indicated that that attribute was not important to parents; however, this did not occur. Attribute levels were derived based on the literature (cost) (Lancsar *et al*. 2022) or expert opinion. The order of attributes was kept consistent between choice tasks.

### Survey design, choice context, and administration

The survey was entered into an online survey platform (Qualtrics). The survey comprised three sections. The first section was an introduction to the choice context and collected participant consent.

The second section entailed the choice tasks. The two block DCE presented each participant with 12 choice tasks. For each choice, task participants were initially asked to choose between two unlabelled alternatives (Initiative 1 or Initiative 2), before them being asked to choose between their initial selection or no initiative at all (example choice task in Supplementary File 2). This method is recommended by Determann *et al*. (2019) to reduce bias.

The final section collected sociodemographic data about the participants and asked attitudinal questions on obesity and climate change. Sociodemographic data were collected to ensure a broad representation of participants and enable subgroup analysis. Categorization of sociodemographic variables was based on Australian Bureau of Statistics surveys (Australian Bureau of Statistics 2019-20, 2022). Sociodemographic parameters collected were age, gender, state of residence, level of education, household income, number of parents/caregivers in the household, number of children 0–17 years in the household, and the number of primary-school-aged children in the household.

Attitudinal questions were included to examine participant views on obesity and climate change. Obesity questions (three questions) were sourced from the literature (Esdaile *et al*. 2021, Ragavan *et al*. 2021), and the climate change questions (three questions) were adapted from the obesity questions. Obesity questions were originally drawn from the questions of The Australian Perceptions of Prevention Survey AUSPOPS, a general population survey which was tested for readability (Grunseit *et al*. 2021). Questions had five-point Likert-scale response options. In addition, participants were asked how confident they felt supporting their child to participate in CBOPIs (five Likert-scale response options ranging from not at all confident to extremely confident). Participants were also asked if other factors would influence their willingness to accept, support, and actively participate in a CBOPI. Responses to this question were not compulsory and were open ended (free text).

### Recruitment

Parents were recruited from all Australian states and territories using a panel company (Pure Profile). Participants were eligible for inclusion if they were a parent or primary caregiver to at least one Australian primary-school-aged child (aged 4–12 years), were aged 18 years or above, lived in Australia, and had internet access and sufficient English language skills. To ensure a sufficient number of participants in each state and territory, quotas were set based on the number of 4- to 12-year olds living in each state and territory (Australian Institute of Health and Welfare 2020, Esdaile *et al*. 2021). Participants received AUD$5.75 from the panel company once they completed the survey.

### Experimental design

A D-efficient design was used to generate an efficient design that would maximize the statistical efficiency (Walker *et al*. 2018). For the initial design, priors were set close to zero and in the anticipated direction (Hensher *et al*. 2015). The survey was piloted twice. The first pilot phase was with a convenience sample of 20 parents and researchers to refine readability and understanding of the requirements of the DCE. In the second pilot stage, the initial design was run with the target population (*n* = 64) to generate priors for the final design. The initial design was estimated setting priors close to zero and in the expected direction. The D-efficient design was re-estimated using the priors from piloting, and the design was conducted with a final sample of 438 parents. The D-efficient design was used as prior values were from a small sample and using priors with a range may have potentially increased standard error (Bliemer and Rose 2005). There is no set guidance for DCE sample size as it depends on the model and population (Bridges *et al*. 2011); however, the average sample size for DCEs in health is 259 with most sample sizes ranging between 100 and 300 (Orme 2006). The final sample size was larger than the average sample size as reported in a systematic review of DCEs in health (*n* = 394) (Nouwens *et al*. 2025) and significantly larger than the proposed guidelines from Johnson and Orme [*n* > 438(12)/(12 × 3) = 146], which were calculated by average number of levels (12), the number of choice tasks (12), and number of alternatives per choice task (3) (de Bekker-Grob *et al*. 2015). Ngene (ChoiceMetrics 2021) was used to generate the design for the pilot and final survey. Stata SE v18.0 (StataCorp, place; StataCorp 2023) was used to check the design for balance.

### Analysis

Nlogit (Econometric Software Plainview USA) (Software 2012) was used for data analysis. Responses were checked for duplication (IP addresses), and straight-line responses (i.e. same response, or response pattern for each question), survey completion, inclusion criteria, and postcodes and states were cross-referenced for consistency.

Cost was coded as a continuous variable, and all other categorical variables were dummy coded. Sociodemographic variables were added to the model if they improved model fit and were statistically significant (*P* < .05).

The final utility function was defined as:

U(Initiative 1) = β_1_Cost + β_2_Aim + β_3_Involvement + β_4_Effectiveness + β_5_Convenience + β_6_social-opportunities

U(Initiative 2) = β_1_Cost + β_2_Aim + β_3_Involvement + β_4_Effectiveness + β_5_Convenience + β_6_social-opportunities

U(Neither) = Neither + β_31_ income$800–1749+ β_32_ income$1750–2999+ β_33_ income$3000^+^ + β_13_ female + β_9_ 34–44years + β_10_ age45–54years

Data were analysed using a conditional logit model with Nlogit (Econometric Software, Plainview, USA). Coefficients, 95% confidence intervals, and *P* values are presented, with significance set at .05. A conditional logit model was used to determine the attribute levels that participants gained the most utility from Hauber *et al*. (2016). A conditional logic models are commonly used in health DCEs (Nouwens *et al*. 2025). For this study, a conditional logit model was selected as the model suited the number of choice tasks and subgroup analysis, and there was limited indication from the initial literature search that that there would be strong preference heterogeneity. A more advanced model was out of scope due to time and resource constraints though it is acknowledged that despite the results of the literature search this may have limited preference heterogeneity. Opt out responses were coded as ‘0’, and participants could select neither option; repeated measures were not included as panel data. Willingness to pay was calculated for each attribute using the Krinsky and Robb method (Hole 2007) in Nlogit with 500 draws. Willingness to Pay (WTP) was used to understand value from the perspective of participants (Grutters *et al*. 2008).

Attitudinal responses were analysed as individual items (Supplementary File 4).

### Results

In total, 613 participants were recorded in April and May 2024. Of these, 438 responses were included in the final analysis sample (71.4%; Table 2). The average completion time was eight minutes (SD = 4 min 23 s). Among the 175 excluded responses, 106 were incomplete, 61 respondents did not meet inclusion criteria based on sociodemographic questions, two were duplicates of included results, and six respondents had completed the survey in <3 minutes [the threshold suggested as too quick by Bansback *et al*. (2014)]. No participants were excluded for straight lining, duplicate IP addresses, or inconsistent postcodes.

Participant demographics are described in Table 1.

**Table 1:** Participant overview.

Characteristic	Count (%) *n* = 438
**Age**	
18–24 years	5 (1.1%)
25–34 years	93 (21.2%)
35–44 years	234 (53.4%)
45–54 years	91 (20.8%)
55+ years	15 (3.4%)
**Gender**	
Male	179 (40.9%)
Female	253 (57.8%)
Nonbinary/third gender	1 (0.2%)
Prefer not to say	5 (1.1%)
**State of residence**	
Australian Capital Territory	9 (2.1%)
New South Wales	137 (31.2%)
Queensland	94 (21.6%)
Victoria	108 (24.5%)
Tasmania	5 (1.2%)
Northern Territory	5 (1.2%)
Western Australia	49 (11.2%)
South Australia	31 (7.1%)
**Education**	
Year 11 or below	25 (5.7%)
Non-school-based qualifications	41 (9.4%)
Year 12 or equivalent	89 (20.3%)
Undergraduate degree	173 (39.5%)
Postgraduate qualification	106 (24.2%)
Prefer not to say	4 (0.9%)
**Household income**	
$0–799/week	37 (8.4%)
$800–1749/week	118 (26.9%)
$1750–2999/week	156 (35.6%)
$3000+	99 (22.6%)
Prefer not to say	28 (6.4%)
**Household composition**	
One parent/caregiver household	57 (13.0%)
Two parent/caregiver household	350 (79.9%)
Multiple family household	20 (4.6%)
Other	3 (0.7%)
Prefer not to say	8 (1.8%)
**Number of children**	
1	106 (24.2%)
2	231 (52.7%)
3	76 (17.4%)
4	20 (4.6%)
5+	5 (1.1%)
**Number of primary-school-aged children**	
1	257 (58.7%)
2	150 (34.3%)
3	23 (5.3%)
4	5 (1.1%)
5+	3 (0.7%)

Parents felt confident to participate in CBOPIs (63.93% felt extremely or very confident to support their child’s participation in CBOPI, and a further 27.63% felt somewhat confident) (Supplementary file 4). Fifty-three respondents reported previously participating in CBOPIs, most commonly community-based physical activity challenges (*n* = 13).

Overall, participants preferred an intervention compared with no intervention (Table 2). The results suggest that parents preferred CBOPIs that aimed to improve both healthy lifestyles and climate change (β = 0.113, *P* = .036). Negative coefficients for cost and convenience suggested that parents preferred CBOPIs that incurred less cost for taxpayers (β = −0.037 *P* = <.001) and that parents were willing to accommodate short-term, manageable disruptions to family schedules in order to participate in CBOPIs (β = −0.145, *P* = <.001). Parents were more likely to prefer interventions that greatly improved healthy lifestyle (β = 0.086, *P* = .053); however, this was not statistically significant. A negative coefficient for convenience suggested parents were willing to accommodate short-term manageable disruptions to family schedules. Female participants (compared with males) and participants with higher household incomes (compared with lower household incomes) were more likely to prefer interventions over no intervention. Participants aged 35–44 years and 45–54 years were less likely to prefer an intervention over no intervention. No significant difference for intervention preferences was found based on education, number of children, number of primary-school-aged children, number of adults or families residing in the household.

**Table 2:** DCE results.

Attributes and demographic factors included in regression model	Coefficient	Standard error	*P* value	95% confidence interval
Initiative cost to the taxpayer (additional cost to you per fortnight, paid as an increase in income taxes)	−0.037	0.008	<.001	−0.05180; −0.02131
Initiative aim Healthy lifestyle (reference) Healthy lifestyle plus climate change	0.113	0.054	.036	0.00758, 0.21779
Initiative involvement Child (reference) You and your child	0.081	0.058	.161	−0.03248, 0.19538
Initiative effectiveness on healthy lifestyle Slightly improves healthy lifestyle (reference) Greatly improves healthy lifestyle	0.086	0.044	.053	−0.00104, 0.17209
Initiative convenience Short term, manageable disruption to family schedule (reference) No interruption to family schedule	−0.145	0.041	<.001	−0.22622; −0.06419
Initiative social opportunities No opportunities for socialization as part of the intervention (reference) Lots of opportunities for socialization as part of the intervention	0.060	0.164	.231	−0.03831; 0.15869
**No intervention** (constant)	−0.412	0.164	.012	−0.73439; −0.09001
Weekly household income $800−$1749 (reference weekly household income $0–$799)	−0.241	0.103	.020	−0.44327; −0.03808
Weekly household income $1750–$2999 (reference weekly household income $0–$799)	−0.550	0.102	<.000	−0.75049; −0.35039
Weekly household income $3000+ (reference weekly household income $0–$799)	−0.333	0.108	.002	−0.54384; −0.12226
Female (reference male)	−0.216	0.069	.002	−0.35103; −0.08129
Age 35–44 years (reference 18–34 years)	0.324	0.089	<.000	0.15036; 0.49818
Age 45–54 years (reference 18–34 years)	0.227	0.103	.027	0.02569; 0.42811

The convenience attribute had the only statistically significant WTP. Parents were willing to pay an additional $3.97 (AUD) per fortnight ($104.000 AUD per annum; Table 3) through additional income tax for CBOPIs that were convenient (*P* = .004). No other attributes had significant WTP results.

**Table 3:** Willingness to pay.

Attribute	Function	Standard error	*P* value	95% confidence interval
Initiative aim	−3.083	2.385	.196	−7.75671; 1.59117
Initiative involvement	−2.228	2.068	.281	−6.28061; 1.82385
Initiative effectiveness on healthy lifestyle	−2.340	1.905	.219	−6.07415; 1.39460
Initiative convenience	3.972	1.347	.003	1.33272; 6.61225

Negative coefficients suggested that parents who were more worried about their child experiencing overweight or obesity or more concerned about the impact of climate change on the health of future generations were less likely to opt out of an initiative (i.e. choose the ‘neither’ option). Conversely, parents who did not believe climate change was a serious concern and parents aged 35–44 years and 45–54 years were more likely to choose the ‘neither’ option to opt out of an initiative.

## Discussion

This study sought to determine the CBOPI preferences of Australian parents with primary-school-aged children. Specifically, it was investigated which factors of CBOPIs parents prefer, and whether parents preferred CBOPIs that aimed to focus on healthy lifestyle or healthy lifestyle and climate change. Identifying the factors that parents prefer may assist in increasing their support, acceptability, and engagement in CBOPIs.

The attributes that were preferred by parents were less cost to taxpayers, high CBOPI effectiveness, and CBOPIs that aimed to include climate change as well as healthy lifestyles. It was clear that convenience was an important attribute for parents. Although they stated willingness to tolerate small, short-term disruptions to family schedules, convenience was the only attribute for which parents had significantly higher WTP. This suggests that convenience was very important to parents and was a key enabler of support, engagement, and participation. This is consistent with the literature, which suggests timing interventions to suit parents’ schedules and supporting parents by offering childcare and food are important intervention strategies to enhance engagement (Walton *et al*. 2018).

Including climate change in CBOPIs was preferred by parents. This is a novel finding as presently climate change is not routinely included as an aim in CBOPIs (Ward *et al*. 2025) and literature on climate change actions in CBOPIs is limited. Though the potential effect size of climate change impacts from CBOPI, it is yet to be established that many CBOPIs have double-duty actions, which may currently only be recognized for their obesity-prevention benefit (Ward *et al*. 2025). There is an opportunity to strengthen both obesity prevention and climate change action with modest modifications to existing interventions, particularly those favoured by parents, which may enhance parent support, engagement, and acceptability. For example, in the Romp & Chomp CBOPI, one of the key messages was improving physical health through reducing sweet beverages (Tran *et al*. 2022). There is an opportunity for this message to be expanded to incorporate environmental benefits, which may impact on parent support for the intervention. Similarly, Ragavan *et al*. (2021) suggests that it would be beneficial to address climate change, similar to other health promotion strategies e.g. sun safety and incorporating double-duty action aims in CBOPIs fits with this recommendation. Increasing the economies of scale of an intervention through the explicit promotion of climate change benefit and obesity prevention may also increase the intervention’s cost-effectiveness. Cost-effectiveness analyses may be used by decision-makers when deciding which interventions to fund (Tran *et al*. 2022). As the outcomes of this study indicate parents prefer CBOPIs that address healthy lifestyle and climate change including climate change objectives in CBOPIs may strengthen parent engagement, support, and acceptability.

Including climate change in the evaluation of CBOPIs may also increase their cost-effectiveness and generate more support from CBOPI decision-makers. A 2023 systematic review on economic evidence for CBOPIs in children found economic evaluations for CBOPIs currently do not include any broader benefits including the potential impacts of climate change (Sultana *et al*. 2023). This identifies an opportunity for further work to investigate how climate change broader benefits can be included in CBOPIs.

Consistent with previous findings (Ragavan *et al*. 2021, Fornwagner and Hauser 2022), most parents indicated that they were concerned about climate change and its impact on their child’s health or the health of future generations. However, parents who did not believe climate change was a concern indicated they would prefer not to engage with an intervention. A 2025 Australian poll on climate action showed strong support for climate action and government (Climate Council 2025). The annual Climate Change and the American Mind study found approximately 70% of Americans believe global warming is happening; however, only 21% understood the scientific consensus amongst climate researchers (Leiserowitz *et al*. 2024). This suggests that whilst there is a general agreement on the adverse effects of climate change, many adults do not have an appreciation of the depth of the scientific evidence. Lawson *et al*. (2019) have proposed that children influence their parents on social topics, meaning that providing intervention on climate change to children may directly influence their parents’ climate change understanding and beliefs.

Parents preferred CBOPIs that cost less to taxpayers. A study by Webb *et al*. (2020) measured WTP for CBOPI decision-makers and similarly found that participants preferred CBOPIs with lower running costs. This was an expected outcome from the results as increases to taxation are not generally viewed favourably.

The effectiveness attribute was not significant at 5%, and the effect size, whilst in the expected direction was small (.086, *P* = .053). This was surprising as it suggests a borderline indifference to the degree of intervention effectiveness in achieving its stated aims. A low sensitivity suggests that perhaps despite survey pretests for comprehension and interpretation, there was not sufficient distinction between ‘slight’ and ‘great’ intervention effect and the delineation between levels of these attributes may have been ambiguous. A DCE requires clear attributes and levels to ensure preferences can be correctly measured (Mangham *et al*. 2009). Alternatively, parents may have considered any small effect to be positive and instead prioritized other attributes such as cost and significance. Lancsar *et al*. (2022) found public support for obesity prevention policies even when they resulted in small health benefits. This was attributed to an attitude that any positive result was beneficial, or perhaps the recognition from participants about broader benefits (e.g. physical, oral health and social opportunities) of obesity prevention (Lancsar *et al*. 2022). The importance of intervention effectiveness for adults who experience overweight and obesity was found by Ryan in their 2014 DCE (Ryan *et al*. 2015) to be an important attribute. In this study, the majority of parents were not concerned about their child experiencing overweight or obesity, so it is reasonable that they did not prioritize achieving substantial effectiveness.

This study found that parents felt confident to support their children in CBOPIs despite the majority of respondents having not previously participated in a CBOPI. This finding is consistent with those from the WAVES study, which found via qualitative interviews that parents were motivated to contribute to CBOPIs (Clarke *et al*. 2015). In the WAVES study, parents wanted to reinforce messages in the home environment, though the level of involvement varied with the level of parent responsibilities (Clarke *et al*. 2015). This suggested that parents felt they had the resources needed to support their child to participate and that additional resourcing as cited in the literature, such as local support staff and additional information sessions, may not be required by most parents (Yavuz *et al*. 2015, Kelleher *et al*. 2017). Further work may need to explore equity considerations and if demographic characteristics are associated with the level of support required.

The attributes for parent involvement and opportunities for socialization did not significantly predict parents’ intervention preferences. This was somewhat unexpected as social opportunities were included after recommendations from the survey pretest. It may be that the attributes were too broad for parents to distinguish. This is consistent with other DCEs (Livingstone *et al*. 2020) and serves to remind CBOPI implementers that CBOPIs need to be contextualized to meet the needs of the local community (Clarke *et al*. 2015). The results of this study suggest that if CBOPI implementers wish to create opportunities for socialization or parental engagement, they should undertake pretesting with the unique intervention community to gauge their specific preferences.

Regarding the subgroup analysis for this study, female respondents were more likely to prefer an intervention and were more likely to be concerned about climate change. Parents who had higher incomes were more likely to prefer an intervention. This suggests that low-income groups may be reluctant to accept increased taxation and/or have other spending priorities. This is interesting as Clarke *et al*. (2015) suggests that households with higher incomes have better health habits and therefore may not benefit as much from CBOPIs, whilst lower income households are more likely to benefit from CBOPI.

### Contribution to broader literature

Parents are a challenging group to engage in CBOPIs (Davison *et al*. 2013, Waters *et al*. 2018); yet, their participation and interest in CBOPIs may enhance intervention outcomes (Davison *et al*. 2013, Hendrie *et al*. 2014). The results of this study may be applied to other studies to enhance parent engagement, acceptability and support.

There is a need to determine which CBOPI factors are most successful in generating positive behaviour change. Future research should focus on whether the incorporation of double-duty actions in CBOPIs generates better intervention outcomes, which double-duty actions offer the greatest benefits and participant acceptability, and what other components are needed in order to increase parent support and engagement in CBOPI.

### Strengths

This novel study had several strengths. To the authors’ knowledge, it is both the first DCE to measure parent CBOPI preferences, and to measure the preference for interventions that aim to reduce obesity or reduce both obesity and climate change. The formative work for the DCE including the literature search, parent involvement in the attribute selection, and piloting was a strength of the study. Parents are a key driver in the success of CBOPIs; however, there are many challenges involved in collecting data from parents (Wang *et al*. 2018). Therefore, extracting parent preferences in a method that can be applied directly to CBOPIs is a strength of the study. CBOPI funders and implementers may incorporate the identified attributes into their work with the intention of increasing parent engagement and support. Additionally, in the absence of participation evidence for other types of community interventions, this evidence may be also applicable to nonobesity community interventions. The use of the panel company enabled recruitment of a geographically representative distribution of participants, from all Australian states and territories.

### Limitations

This study adds to the small volume of current literature on influences on parent engagement, support, and acceptability of CBOPIs. Therefore, the number of attributes available to be identified in the literature was sparse. Although this was addressed by investment in pilot testing and asking participants to identify additional motivators, there are possibly attitudinal attributes that were not measured. Double-duty actions have potential effect on obesity and climate change; however, their impact in CBOPIs has not been confirmed. The study focuses on how parent attitudes may shift if climate change action was part of CBOPI messaging; however, the impact of climate change action from CBOPI is not explored. There is also potential for hypothetical bias despite the anonymous survey. Preference data may also be influenced by the data distribution and context of interpretation. Whilst measures were in place to mitigate these risks including a significant sample size, these may influence results. Conditional logit was selected as the analysis model as it could determine the characteristics of the CBOPI; however, conditional logit models do not account for scale heterogeneity or preference heterogeneity (Hauber *et al*. 2016). More sophisticated models could be explored in further research, to account for preference differences among respondents. There were no significant results for household composition and education level. This may be also explored in further research. Additionally, as the inclusion criteria required English language proficiency and internet access the results may introduce bias for parents who do not meet these criteria.

## Conclusions

This study demonstrated that parents prefer CBOPIs that include healthy lifestyles and climate change. This contributes to the evidence that there are opportunities for CBOPIs to include double-duty actions. This information can assist to ensure CBOPIs are designed to generate maximum parent acceptability, support, and engagement. The preference of including climate change in CBOPIs may assist to simultaneously promote and address two of the greatest public health concerns, obesity and climate change.

## Supplementary Material

daag033_Supplementary_Data

## Data Availability

Datasets are securely stored on a Deakin University password protected drive and may be made available on request. The data underlying this article will be shared on reasonable request to the corresponding author.

## Statements and declarations

For submission as an original article.

## References

[daag033-B1] Allender S, Millar L, Hovmand P et al Whole of systems trial of prevention strategies for childhood obesity: WHO STOPS childhood obesity. Int J Environ Res Public Health 2016;13:1143. 10.3390/ijerph13111143 27854354 PMC5129353

[daag033-B2] Australian Bureau of Statistics . Household Income and Wealth, Australia. Canberra: Australian Bureau of Statistics, 2019-20. https://www.abs.gov.au/statistics/economy/finance/household-income-and-wealth-australia/2019-20

[daag033-B3] Australian Bureau of Statistics . National Health Survey. Canberra: Australian Bureau of Statistics, 2022. https://www.abs.gov.au/statistics/health/health-conditions-and-risks/national-health-survey/latest-release

[daag033-B4] Australian Institute of Health and Welfare . Australia's Children. Canberra: Australian Institute of Health and Welfare, 2020. https://www.aihw.gov.au/getmedia/6af928d6-692e-4449-b915-cf2ca946982f/aihw-cws-69-print-report.pdf.aspx?inline=true

[daag033-B5] Bansback N, Hole AR, Mulhern B et al Testing a discrete choice experiment including duration to value health states for large descriptive systems: addressing design and sampling issues. Soc Sci Med 2014;114:38–48. 10.1016/j.socscimed.2014.05.026 24908173 PMC4074344

[daag033-B6] Bleich SN, Segal J, Wu Y et al Systematic review of community-based childhood obesity prevention studies. Pediatrics 2013;132:e201–10. 10.1542/peds.2013-0886 23753099 PMC3691541

[daag033-B7] Bliemer MCJ, Rose JM. Efficiency and Sample Size Requirements for Stated Choice Studies. Sydney: The University of Sydney, 2005.

[daag033-B8] Bridges JF, Hauber AB, Marshall D et al Conjoint analysis applications in health–a checklist: a report of the ISPOR Good Research Practices for Conjoint Analysis Task Force. Value Health 2011;14:403–13. 10.1016/j.jval.2010.11.013 21669364

[daag033-B9] Brown T, Moore TH, Hooper L et al Interventions for preventing obesity in children. Cochrane Database Syst Rev 2019;7:CD001871. 10.1002/14651858.CD001871.pub4 31332776 PMC6646867

[daag033-B10] Bryant M, Burton W, Cundill B et al Effectiveness of an implementation optimisation intervention aimed at increasing parent engagement in HENRY, a childhood obesity prevention programme—the Optimising Family Engagement in HENRY (OFTEN) trial: study protocol for a randomised controlled trial. Trials 2017;18:40. 10.1186/s13063-016-1732-3 28115006 PMC5260000

[daag033-B11] ChoiceMetrics . Ngene. 1.3. 2021. https://www.choice-metrics.com/

[daag033-B12] Clarke JL, Griffin TL, Lancashire ER et al Parent and child perceptions of school-based obesity prevention in England: a qualitative study. BMC Public Health 2015;15:1224. 10.1186/s12889-015-2567-7 26654046 PMC4674916

[daag033-B13] Climate Council . *New Poll Shows Aussies Back Strong Climate Action, as Unnatural Disasters Dent Productivity*. 2025. https://www.climatecouncil.org.au/resources/new-poll-shows-aussies-back-strong-climate-action-as-unnatural-disasters-dent-productivity/ (November 2025, date last accessed).

[daag033-B14] Davison K, Jurkowski J, Li K et al A childhood obesity intervention developed by families for families: results from a pilot study. Int Behav Nutr Phys Act 2013;10:3. 10.1186/1479-5868-10-3 PMC3547740 23289970

[daag033-B15] de Bekker-Grob EW, Donkers B, Jonker MF et al Sample size requirements for discrete-choice experiments in healthcare: a practical guide. Patient 2015;8:373–84. 10.1007/s40271-015-0118-z 25726010 PMC4575371

[daag033-B16] Determann D, Gyrd-Hansen D, de Wit GA et al Designing unforced choice experiments to inform health care decision making: implications of using opt-out, neither, or Status quo alternatives in discrete choice experiments. Med Decis Making 2019;39:681–92. 10.1177/0272989X19862275 31354031

[daag033-B17] Esdaile E, Owen KB, Xu H et al Strong support for broad policies to prevent childhood obesity among mothers in New South Wales, Australia. Health Promot J Austr 2021;32:197–207. 10.1002/hpja.351 32333441

[daag033-B18] Fornwagner H, Hauser OP. Climate action for (my) children. Environ Resour Econ (Dordr) 2022;81:95–130. 10.1007/s10640-021-00620-7 34803223 PMC8593181

[daag033-B19] Grunseit AC, Howse E, Bohn-Goldbaum E et al Changes in Australian community perceptions of non-communicable disease prevention: a greater role for government? BMC Public Health 2021;21:2094. 10.1186/s12889-021-12159-9 34781923 PMC8591602

[daag033-B20] Grutters JP, Kessels AG, Dirksen CD et al Willingness to accept versus willingness to pay in a discrete choice experiment. Value Health 2008;11:1110–9. 10.1111/j.1524-4733.2008.00340.x 18489505

[daag033-B21] Hauber AB, Gonzalez JM, Groothuis-Oudshoorn CG et al Statistical methods for the analysis of discrete choice experiments: a report of the ISPOR Conjoint Analysis Good Research Practices Task Force. Value Health 2016;19:300–15. 10.1016/j.jval.2016.04.004 27325321

[daag033-B22] Hendrie GA, Ridoutt BG, Wiedmann TO et al Greenhouse gas emissions and the Australian diet–comparing dietary recommendations with average intakes. Nutrients 2014;6:289–303. 10.3390/nu6010289 24406846 PMC3916862

[daag033-B23] Hensher DA, Rose JM, Greene WH. Applied Choice Analysis, 2nd edn. Cambridge: Cambridge University Press, 2015.

[daag033-B24] Hole AR . A comparison of approaches to estimating confidence intervals for willingness to pay measures. Health Econ 2007;16:827–40. 10.1002/hec.1197 17238222

[daag033-B25] Jurkowski JM, Green Mills LL, Lawson HA et al Engaging low-income parents in childhood obesity prevention from start to finish: a case study. J Community Health 2013;38:1–11. 10.1007/s10900-012-9573-9 22714670 PMC3547242

[daag033-B26] Kelleher E, Harrington JM, Shiely F et al Barriers and facilitators to the implementation of a community-based, multidisciplinary, family-focused childhood weight management programme in Ireland: a qualitative study. BMJ Open 2017;7:e016459. 10.1136/bmjopen-2017-016459 PMC5623413 28851786

[daag033-B27] Lancsar E, Fiebig DG, Hole AR. Discrete choice experiments: a guide to model specification, estimation and software. Pharmacoeconomics 2017;35:697–716. 10.1007/s40273-017-0506-4 28374325

[daag033-B28] Lancsar E, Ride J, Black N et al Social acceptability of standard and behavioral economic inspired policies designed to reduce and prevent obesity. Health Econ 2022;31:197–214. 10.1002/hec.4451 34716628 PMC9298376

[daag033-B29] Lawson DF, Stevenson KT, Peterson MN et al Children can foster climate change concern among their parents. Nat Clim Change 2019;9:458–62. 10.1038/s41558-019-0463-3

[daag033-B30] Leiserowitz A, Maibach E, Rosenthal S et al Climate Change in the American Mind: Beliefs & Attitudes. Yale Program on Climate Change Communication, Yale University and George Mason University. New Haven, CT: Spring, 2024.

[daag033-B31] Livingstone KM, Lamb KE, Abbott G et al Ranking of meal preferences and interactions with demographic characteristics: a discrete choice experiment in young adults. Int J Behav Nutr Phys Act 2020;17:157. 10.1186/s12966-020-01059-7 33261647 PMC7708905

[daag033-B32] Mangham LJ, Hanson K, McPake B. How to do (or not to do) … Designing a discrete choice experiment for application in a low-income country. Health Policy Plan 2009;24:151–8. 10.1093/heapol/czn047 19112071

[daag033-B33] Nouwens SPH, Marceta SM, Bui M et al The evolving landscape of discrete choice experiments in health economics: a systematic review. Pharmacoeconomics 2025;43:879–936. 10.1007/s40273-025-01495-y 40397369 PMC12255651

[daag033-B34] Orme B . Getting Started with Conjoint Analysis: Strategies for Product Design and Pricing Research. Manhattan Beach, CA: Research Publishers, 2006.

[daag033-B35] Ragavan MI, Marcil LE, Philipsborn R et al Parents’ perspectives about discussing climate change during well-child visits. J Clim Change Health 2021;4:100048. 10.1016/j.joclim.2021.100048

[daag033-B36] Ride J, Goranitis I, Meng Y et al A reporting checklist for discrete choice experiments in health: the DIRECT checklist. Pharmacoeconomics 2024;42:1161–75. 10.1007/s40273-024-01431-6 39227559 PMC11405421

[daag033-B37] Ryan M, Yi D, Avenell A et al Gaining pounds by losing pounds: preferences for lifestyle interventions to reduce obesity. Health Econ Policy Law 2015;10:161–82. 10.1017/S1744133114000413 25348049

[daag033-B38] Smith KL, Straker LM, McManus A et al Barriers and enablers for participation in healthy lifestyle programs by adolescents who are overweight: a qualitative study of the opinions of adolescents, their parents and community stakeholders. BMC Pediatr 2014;14:53. 10.1186/1471-2431-14-53 24552207 PMC3942615

[daag033-B39] Soekhai V, de Bekker-Grob EW, Ellis AR et al Discrete choice experiments in health economics: past, present and future. Pharmacoeconomics 2019;37:201–26. 10.1007/s40273-018-0734-2 30392040 PMC6386055

[daag033-B40] Software E . NLOGIT. United States of America. 2012. https://apps.econ.cmu.ac.th/econ_sw/?page_id=119 (June to August 2024, date last accessed).

[daag033-B41] StataCorp . Stata Statistical Software: Release 18. College Station, TX: StataCorp LLC, 2023.

[daag033-B42] St George SM, Messiah SE, Sardinas KM et al Familias unidas for health and wellness: adapting an evidence-based substance use and sexual risk behavior intervention for obesity prevention in Hispanic adolescents. J Prim Prev 2018;39:529–53. 10.1007/s10935-018-0524-9 30291486 PMC6585459

[daag033-B43] Sultana M, Nichols M, Jacobs J et al The range of outcomes and outcome measurement instruments collected in multisectoral community-based obesity prevention interventions in children: a systematic review. Obes Rev 2024;25:e13731. 10.1111/obr.13731 38432682

[daag033-B44] Sultana M, Nichols M, Moodie M et al A systematic review of economic evidence for community-based obesity prevention interventions in children. Obes Rev 2023;24:e13592. 10.1111/obr.13592 37308321 PMC10909472

[daag033-B45] Swinburn BA, Kraak VI, Allender S et al The global syndemic of obesity, undernutrition, and climate change: the Lancet Commission report. The Lancet 2019;393:791–846. 10.1016/S0140-6736(18)32822-8 30700377

[daag033-B46] Tran HNQ, Killedar A, Tan EJ et al Cost-effectiveness of scaling up a whole-of-community intervention: the Romp & Chomp early childhood obesity prevention intervention. Pediatr Obes 2022;17:e12915. 10.1111/ijpo.12915 35301814 PMC9540361

[daag033-B47] Walker JL, Wang Y, Thorhauge M et al D-efficient or deficient? A robustness analysis of stated choice experimental designs. Theory Decis 2018;84:215–38. 10.1007/s11238-017-9647-3

[daag033-B48] Walton K, Ambrose T, Annis A et al Putting family into family-based obesity prevention: enhancing participant engagement through a novel integrated knowledge translation strategy. BMC Med Res Methodol 2018;18:126. 10.1186/s12874-018-0588-5 30409164 PMC6225617

[daag033-B49] Wang Z, Xu F, Ye Q et al Childhood obesity prevention through a community-based cluster randomized controlled physical activity intervention among schools in China: The Health Legacy Project of the 2nd World Summer Youth Olympic Games (YOG-Obesity study). Int J Obes 2018;42:625–33. 10.1038/ijo.2017.243 PMC5984083 28978975

[daag033-B50] Ward N, Nichols M, Moodie M et al Are climate-change actions present in community-based obesity prevention interventions? Development and application of the DoublE-duty actions in CommunIty-baSed obesity InterVEntions (DECISIVE) framework. J Public Health 2025;33:2093–104. 10.1007/s10389-023-02177-9

[daag033-B51] Waters E, Gibbs L, Tadic M et al Cluster randomised trial of a school-community child health promotion and obesity prevention intervention: findings from the evaluation of fun ‘n healthy in Moreland!. BMC Public Health 2018;18:92. 10.1186/s12889-017-4625-9 PMC5543738 28774278

[daag033-B52] Webb EJD, Stamp E, Collinson M et al Measuring commissioners’ willingness-to-pay for community based childhood obesity prevention programmes using a discrete choice experiment. BMC Public Health 2020;20:1535. 10.1186/s12889-020-09576-7 33046078 PMC7549208

[daag033-B53] White AA, Colby SE, Franzen-Castle L et al The iCook 4-H Study: an intervention and dissemination test of a youth/adult out-of-school program. J Nutr Educ Behav 2019;51:S2–20. 10.1016/j.jneb.2018.11.012 30851861

[daag033-B54] Yavuz HM, van Ijzendoorn MH, Mesman J et al Interventions aimed at reducing obesity in early childhood: a meta-analysis of programs that involve parents. J Child Psychol Psychiatry 2015;56:677–92. 10.1111/jcpp.12330 25292319

[daag033-B55] Zeb A, Sivarajan Froelicher E, Pienaar AJ et al Effectiveness of community-based obesity intervention for body weight, body mass index, and waist circumference: meta-analysis. Iran J Nurs Midwifery Res 2024;29:16–22. 10.4103/ijnmr.ijnmr_120_22 38333330 PMC10849288

