# Supplementary material for: Valuing Australian parent preferences for community-based nutrition and physical activity initiatives: a discrete choice experiment
Source: Health Promot Int. 2026 Mar 9;41(2):daag033. doi: 10.1093/heapro/daag033 (PMC13017149; doi:10.1093/heapro/daag033)

**Discrete Choice Experiment Survey**

**Understanding parent preferences for community-based healthy lifestyle initiatives**

Healthy lifestyle initiatives aim to encourage and support healthy eating and active living in children.

Community-based healthy lifestyle initiatives might include things like healthy canteen policies or walk to school days. In addition to an impact on healthy eating and active living, some of these initiatives might also impact climate change.

In this study, we would like to know if parents prefer **healthy lifestyle initiatives that solely focus on healthy eating and active living**; or if parents prefer healthy lifestyle initiatives that focus on**both healthy eating and active living and climate change**.

**You will be presented with 12 'choice tasks'**. In each of these, some basic features of two healthy lifestyle initiatives will be described. You will be asked to choose the one that you as a parent most prefer based on the information given.

The survey should take no longer than 15-20 minutes to complete. Your answers will help us to better understand which factors help to make healthy lifestyle initiatives most successful.

Thank you for your participation. This study is being undertaken by researchers at Deakin University and Monash University. The Deakin Human Research Ethics Committee (HREC) has approved this study (HEAG-H 11_2024). If you have any questions or would like further information, please contact Nicole Ward: nicole.ward@deakin.edu.au.

Language Statement and Consent

Important information on your participation in our study is available in the downloadable [Plain language statement](https://researchsurveys.deakin.edu.au/CP/File.php?F=F_85GgRi3048ehqaG)**.** This information explains the study procedure and how your data will be used and stored. Please take the time to download and read this information.

Have you read and understood the Plain Language Statement and do you agree to take part in this project according to the conditions described?

Yes, I agree to take part according to the conditions in the Plain Language Statement and acknowledge that the researcher has agreed not to reveal my identity and personal details, including where information about this project is published, or presented in any public form.

No, I do not agree to participate.

Before you are directed to the study survey, please answer the following two questions to ensure you are eligible to participate.

Are you aged over 18 years, a parent/caregiver of at least one primary-school aged child (aged 4-12 years), and a resident in Australia and comfortable reading English?

Yes

No

What state/territory do you currently reside in? [forced choice, dropdown menu]

- ACT (1)
- NSW (2)
- QLD (3)
- VIC (4)
- TAS (5)
- NT (6)
- WA (7)
- SA (8)

[If quota reached for state/territory – Skip logic to ‘end survey block-reached quota’]

The survey has three sections.

**Section A** consists of the “choice task”. Features of two healthy lifestyle initiatives are described.
You are asked to choose which of the two initiatives you most prefer.
Please choose the one you would most prefer to be available as an initiative for your child in your community. After you have selected which of the two options you most prefer, you will then be asked if you would prefer the initiative you chose or no initiative.
You will complete this task 12 times.

**Section B** will ask some questions about your views on both obesity and climate change.

**Section C** will ask some questions about you and your household.

Thank you for participating in this study.

Community-based healthy lifestyle initiatives aim to improve healthy eating and/or active living in children. They occur in community settings, like schools, recreational facilities and neighbourhoods.

Initiatives may also have the potential to improve climate change. An example might be walk to school days, where walking or riding a bike might replace car travel. Another example might be a “water-only” policy at recreational centres, where water might replace sugary drinks. These are good for children's health, and also for the environment.

Community-based healthy lifestyle initiatives might vary by:

-The annual cost of the initiative. Costs might vary depending on the type of initiative, but generally they are publicly funded i.e. paid for by the government through taxation and other revenue.

- The aim of the initiative. The initiative may aim to improve healthy lifestyles (healthy eating and/or active living), or it might aim to improve both healthy lifestyles and climate change.

-Initiative involvement. The initiative may involve you and your child by engaging you both in activities, education sessions, or by following policies. Or the initiative may only involve your child.

- Initiative effectiveness. For example, the initiative may have no effect on healthy lifestyle or it may positively improve healthy lifestyle.

- Initiative convenience. The initiative may have no interruption to family schedule or it may cause a short term, manageable disruption to family schedule.

- Initiative opportunity for social interaction. The initiative may have lots of opportunities for social interaction through shared activities or education session, or the initiative may have no opportunities for social interaction.

Next we will go through an example of the "choice task".


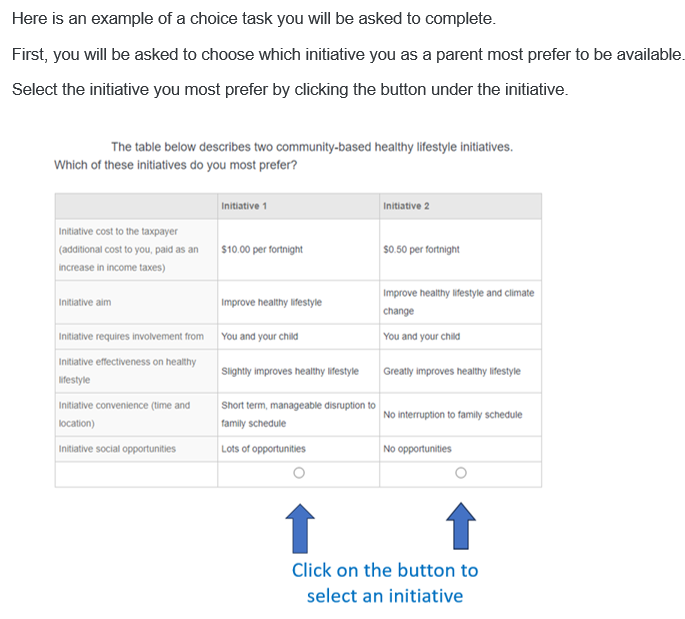


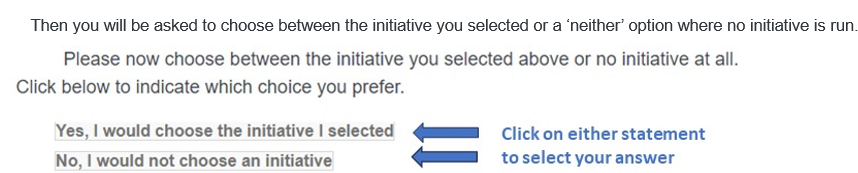


Here is a [Choice task guide](https://researchsurveys.deakin.edu.au/CP/File.php?F=F_4PLFdY3IriumXA2) (click to open in a new window) with extra information to help you select the one you would most prefer.
After you read it remember to come back and complete the survey.

Click the "next" button to start the choice tasks.

[Conjoint question appear here]

Healthy lifestyle initiatives may help to reduce or prevent obesity. We would now like to hear your views on the importance of healthy lifestyle initiatives and obesity prevention.

Please respond to the following questions or statements.
How worried are you about your child experiencing overweight or obesity?

- Not worried at all
- A little bit worried
- Moderately worried
- Very worried
- Extremely worried

How worried should parents be about their child experiencing overweight or obesity?

- Not worried at all
- A little bit worried
- Moderately worried
- Very worried
- Extremely worried

Childhood obesity is a serious public health issue.

- Strongly disagree
- Somewhat disagree
- Neither agree nor disagree
- Somewhat agree
- Strongly agree

We would now like to hear your views on the importance of healthy lifestyle initiatives and climate change.

Please respond to the following questions or statements.
Climate change is a serious concern.

- Strongly disagree
- Somewhat disagree
- Neither agree nor disagree
- Somewhat agree
- Strongly agree

How worried should parents be about climate change impacting on their child’s health?

- Not worried at all
- A little bit worried
- Moderately worried
- Very worried
- Extremely worried

I am concerned about the impact of climate change on future generations.

- Strongly disagree
- Somewhat disagree
- Neither agree nor disagree
- Somewhat agree
- Strongly agree

How confident do you feel to support your child in participating in a healthy lifestyle initiative?

- Not at all confident
- Slightly confident
- Somewhat confident
- Very confident
- Extremely confident

Please answer the following demographic questions. You will not be asked to provide any information which could be used to identify you.

What is your age?

- 18-24 yrs
- 25-34 yrs
- 35-44 yrs
- 45-54 yrs
- 55+ yrs

What is your gender identity?

- Male
- Female
- Non-binary/ third gender
- Prefer not to say

What is your postcode?

What is your highest education qualification?

- Year 11 or below
- Non-school based qualification e.g. Trade certificate
- Year 12 or equivalent
- Undergraduate degree
- Post-graduate qualification
- Prefer not to say

What is your average weekly household income before tax? This is the total income of all people who live in your household.

- $0-799 per week
- $800-$1749 per week
- $1750-$2999 per week
- $3000 or above
- Prefer not to say

Which statement below best describes your household.

- One parent/ caregiver household
- Two parent/ caregiver household
- Multiple family household
- Other
- Prefer not to say

How many children (aged 0-17 years) usually live in your household?
"Usually live" means the persons considers this their home or primary place of residence.

How many primary school aged children (aged 4-12 years) usually live in your household?
"Usually live" means the child considers this their home or primary place of residence.

Which other factors influence your willingness to accept, support, and actively participate in initiatives that promote a healthy lifestyle?

 Have you previously participated in a healthy lifestyle initiative?
[Next question skipped if no is selected]

- Yes
- No
- Unsure

Please provide details of any healthy lifestyle initiative you have participated in.

Thank you for your interest in our study. We appreciate the time you have taken today. If you would like to be informed of the results of this study, please provide an email address that we can send study results to. For further information, please contact Nicole Ward, Deakin University, nicole.ward@deakin.edu.au

Discrete Choice Experiment instrument, Study3

The 12 choice tasks are presented below. As illustrated after the first choice task participants were asked after each selection if they would select their choice of initiative or no initiative at all.


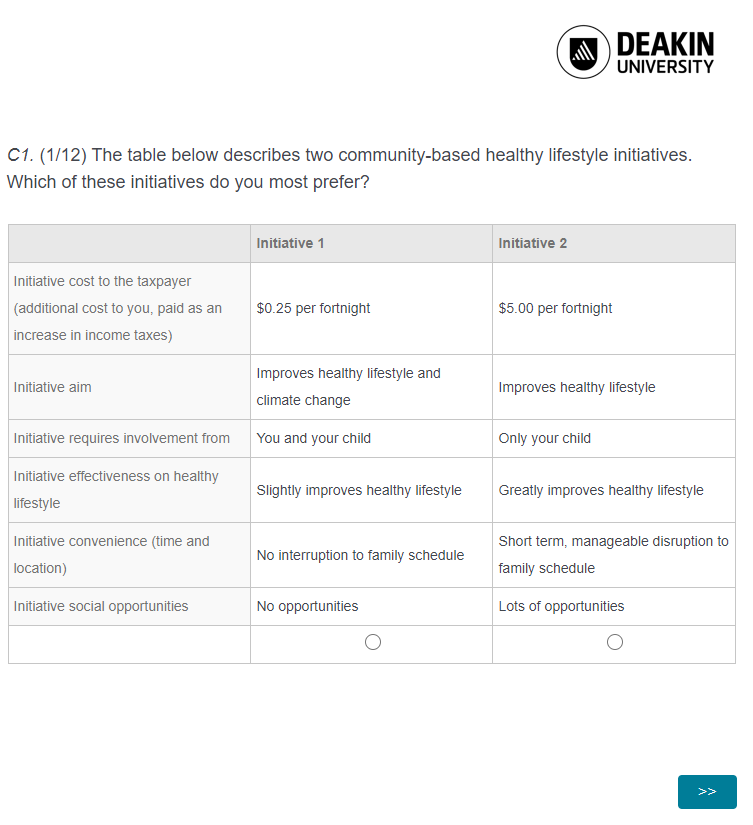


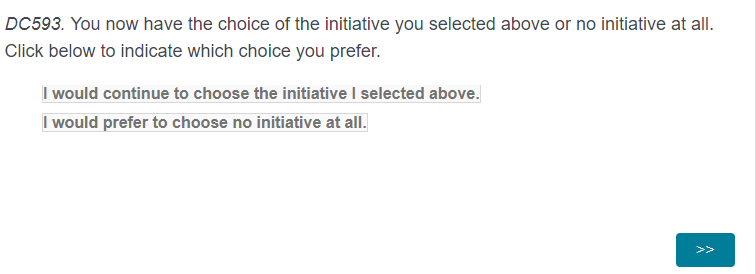


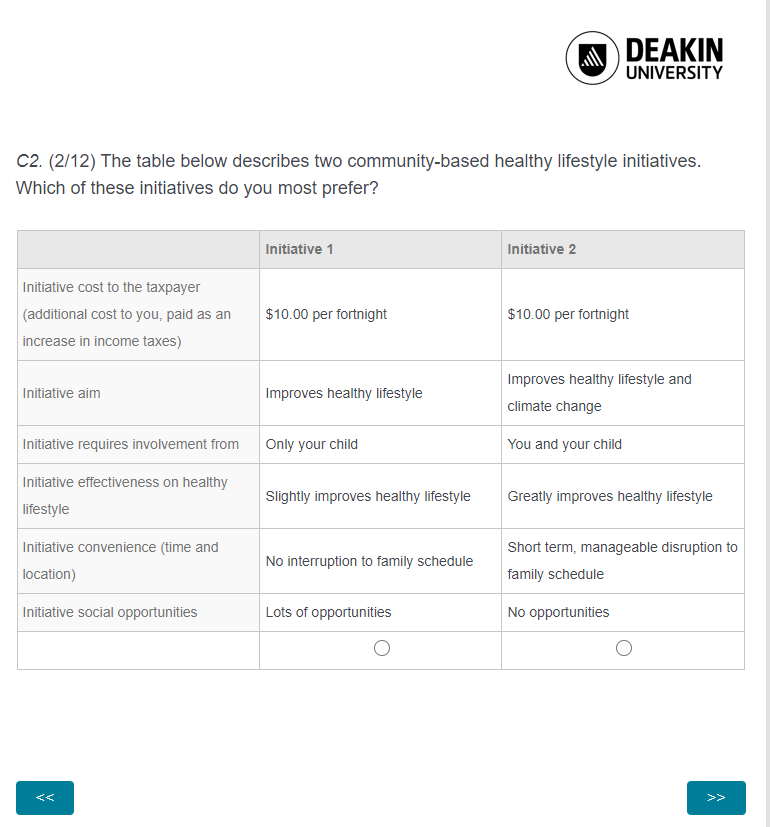


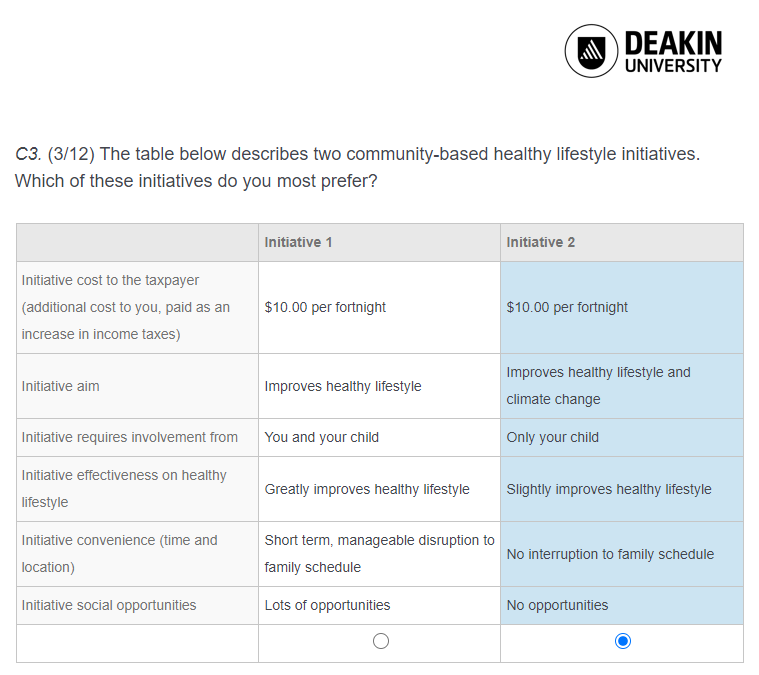


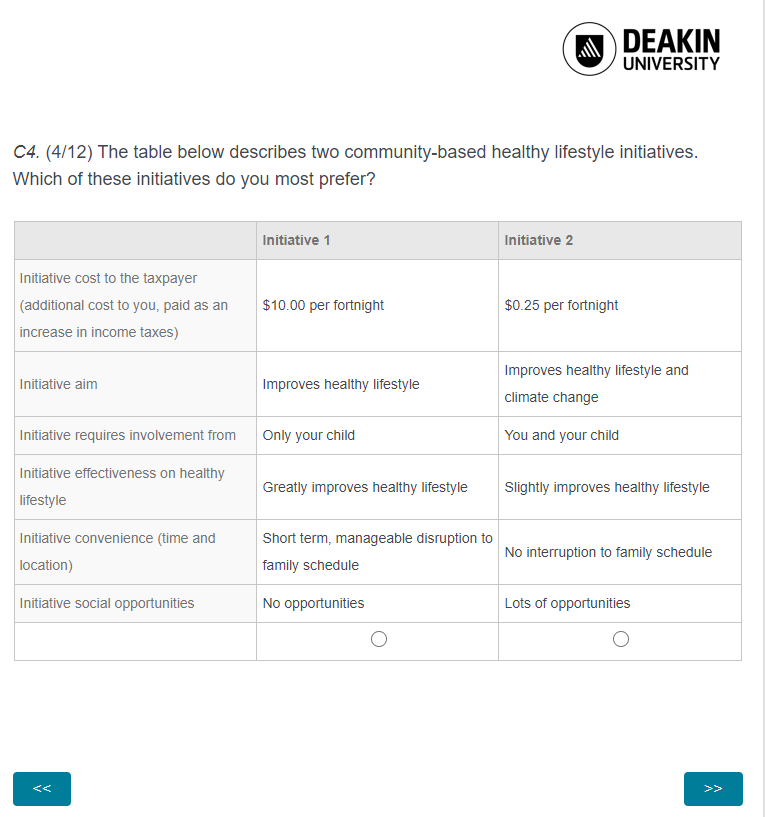


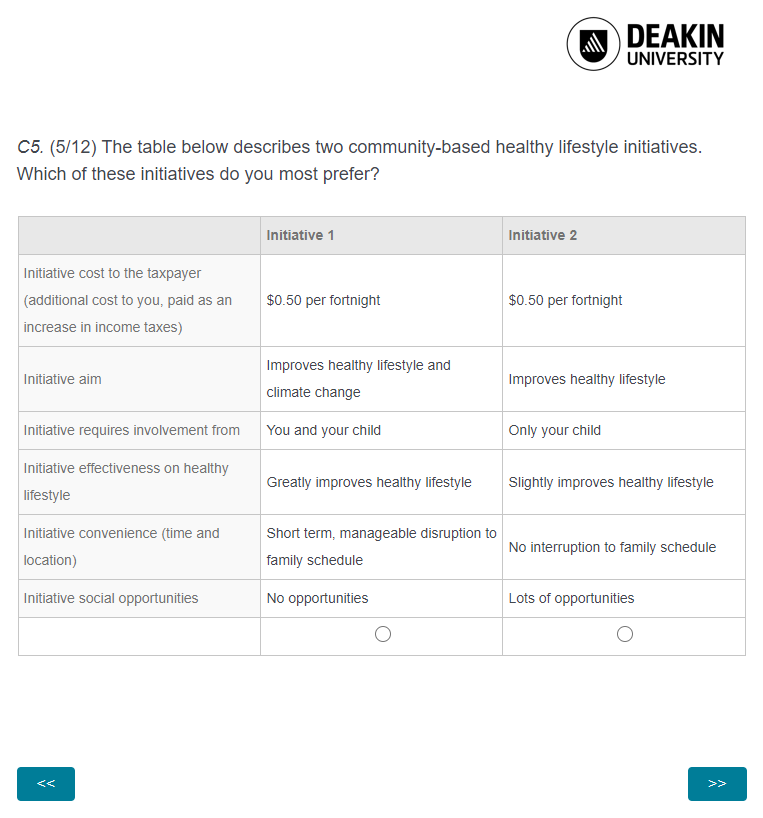


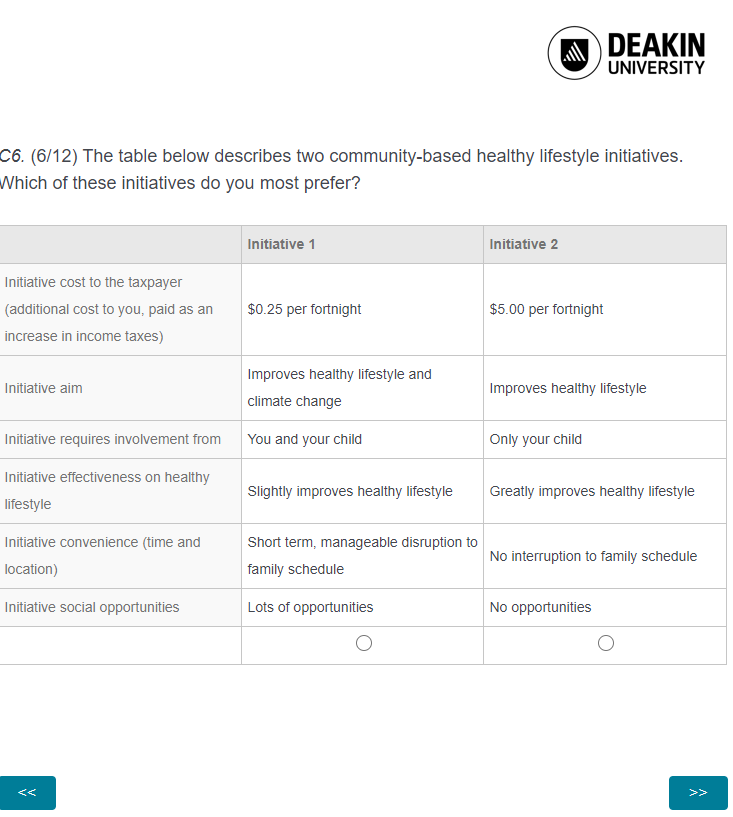


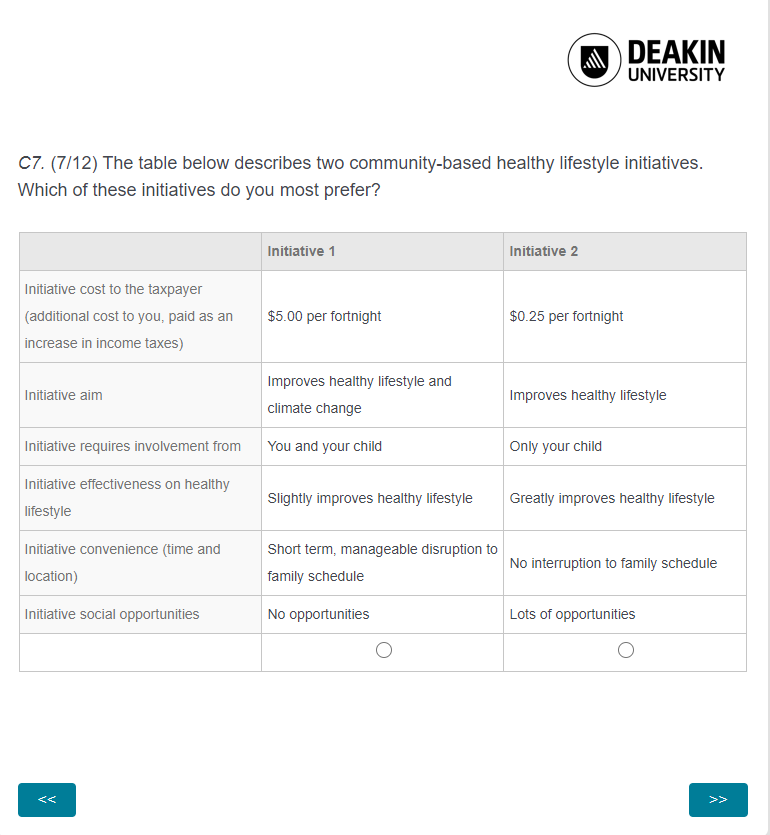


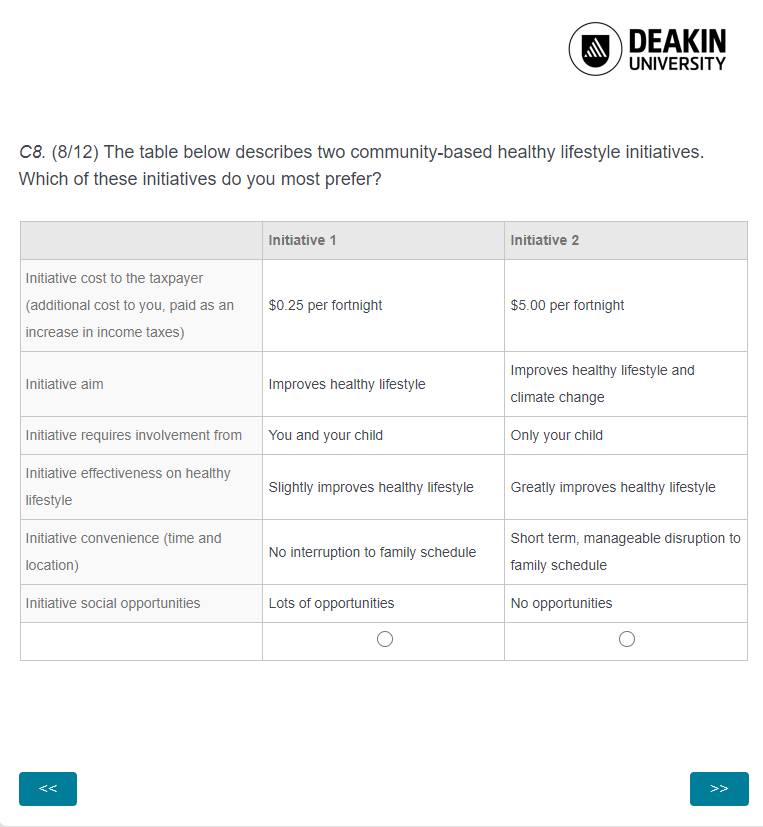


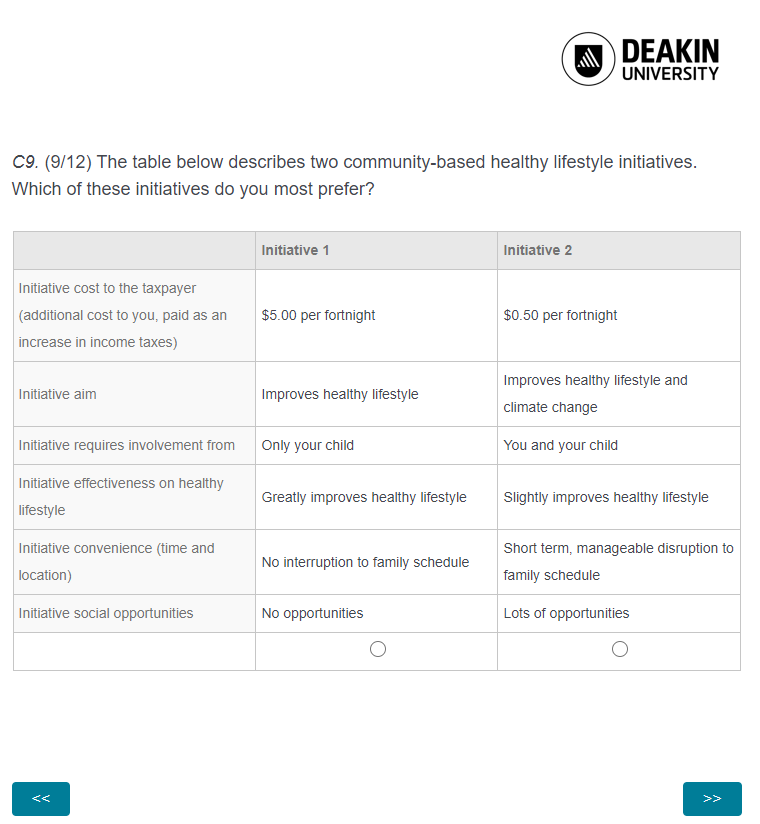


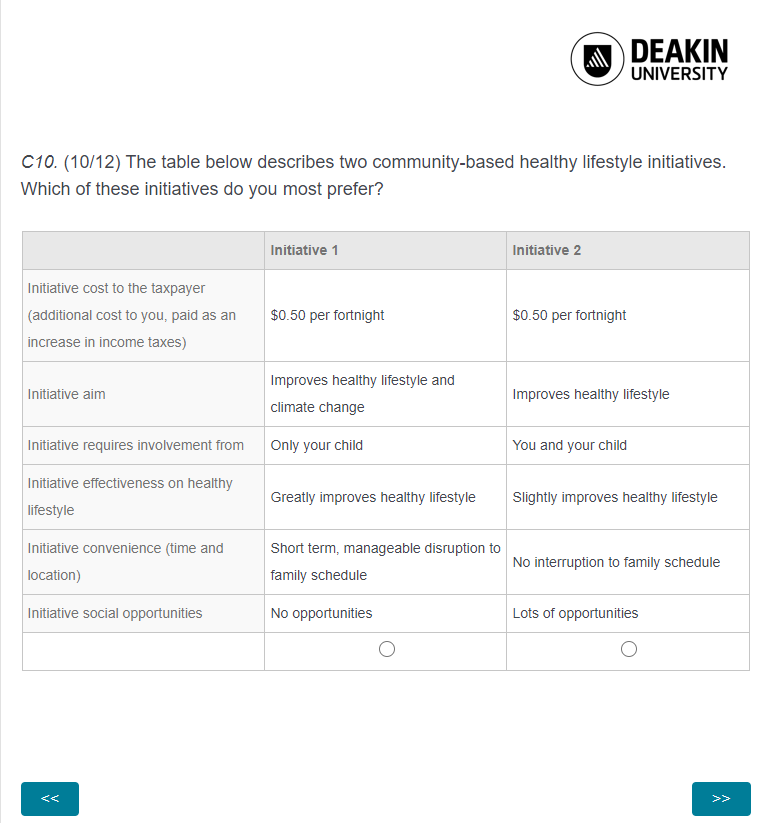


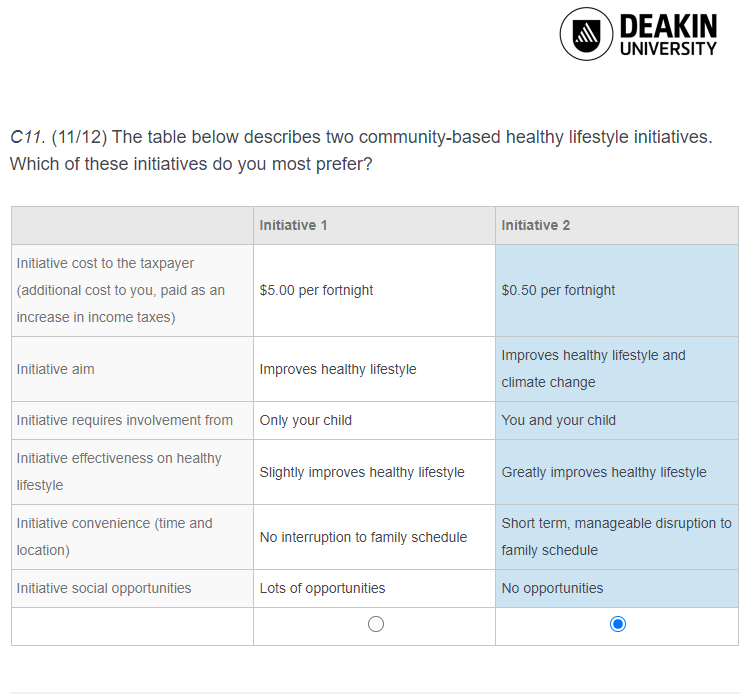


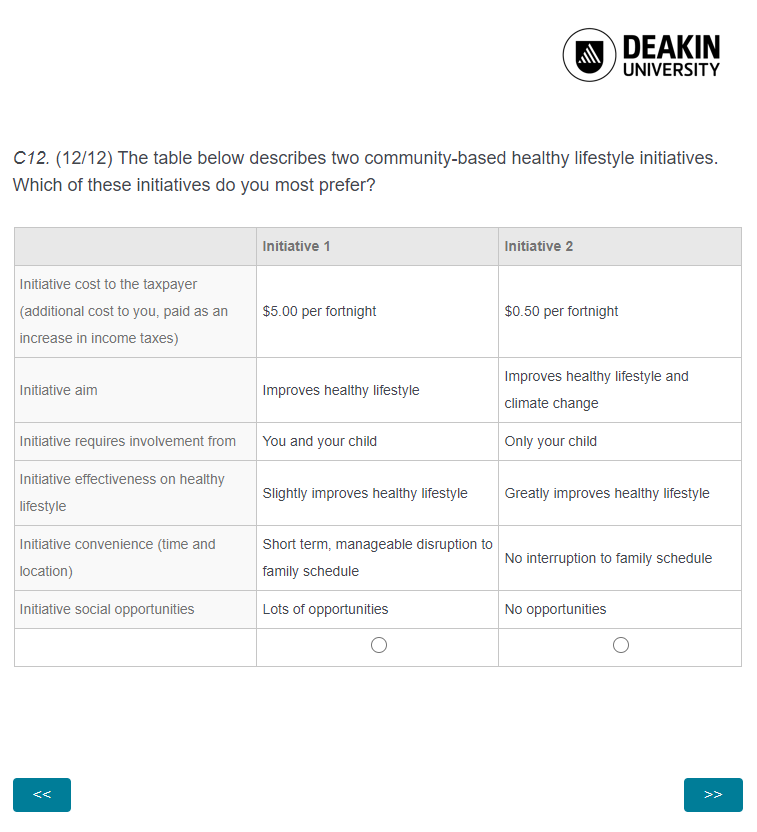

Supplement: daag033_Supplementary_Data [file daag033_supplementary_data.zip › Supplementary file 3 Discrete Choice Experiment Survey.docx]
