# Supplementary material for: Valuing Australian parent preferences for community-based nutrition and physical activity initiatives: a discrete choice experiment
Source: Health Promot Int. 2026 Mar 9;41(2):daag033. doi: 10.1093/heapro/daag033 (PMC13017149; doi:10.1093/heapro/daag033)
Supplement: daag033_Supplementary_Data [file daag033_supplementary_data.zip › Supplementary File 1 Potential attributes.docx]

Supplementary File 1 Potential attributes

| **Attribute** | **Included/ excluded** | **Reason** |
| --- | --- | --- |
| Parents enjoyed time with their children ^[1]^ | Included | Currently unknown |
| Parents and children involved / family-based intervention ^[1-4]^ | Included | Currently unknown. |
| Motivated by topic ^[5]^ | Included | Currently unknown.  Topic refined to answer research question. |
| Social connection ^[5]^ | Included | Currently unknown. |
| Convenient timing ^[5, 6]^ | Included | Currently unknown. |
| Location convenient ^[6]^ | Included | Currently unknown |
| Dyads involved in design^[1]^ | Excluded | Not relevant to all CBOPIs |
| Cash/ gift card incentive ^[1, 6]^ | Excluded | Participants paid to participate in CBOPI. May skew results. |
| Practical, engaged sessions ^[1, 5, 7]^ | Excluded | Deemed to be existing attribute of CBOPIs based on expert opinion |
| Effective, supportive program leaders ^[1, 4]^ | Excluded | Deemed to be existing attribute of CBOPIs based on expert opinion |
| Run in the community ^[1]^ | Excluded | Deemed to be existing attribute of CBOPIs based on expert opinion |
| Bilingual facilitator ^[5]^ | Excluded | Not relevant to all communities. |
| Project coordinator ^[6]^ | Excluded | Deemed to be existing attribute of CBOPIs based on expert opinion. |
| Childcare provided ^[6]^ | Excluded | Not relevant/ practical for all CBOPIs. |
| Food provided ^[6]^ | Excluded | Not relevant/ practical for all CBOPIs. |
| Activities for parents ^[7]^ | Excluded | Combined into parent child activities. |
| Siblings needs ^[4]^ | Excluded | Out of scope. |
| Lack of space at home ^[4]^ | Excluded | Out of scope. |
| Competing demands on family ^[4]^ | Excluded | Out of scope. |
| Perceived direct costs of participating ^[4]^ | Excluded | CBOPI costs paid by tax payer |
| Messages reiterated at school ^[4]^ | Excluded | Relevance dependent on type of CBOPI. |

Table notes: Included attributes =

1. White, A.A., Colby, S.E., Franzen-Castle, L., et al., *The iCook 4-H Study: An intervention and dissemination test of a youth/adult out-of-school program.* Journal of Nutrition Education and Behavior, 2019. **51**(3, Suppl): p. S2-S20.

2. Davison, K., Jurkowski, J., Li, K., et al., *A childhood obesity intervention developed by families for families: results from a pilot study.* International Journal of Behavioural Nutrition and Physical Actvity, 2013. **10**(3).

3. Brown, T., Moore, T.H., Hooper, L., et al., *Interventions for preventing obesity in children.* Cochrane Database Syst Rev, 2019. **7**: p. CD001871.

4. Clarke, J.L., Griffin, T.L., Lancashire, E.R., et al., *Parent and child perceptions of school-based obesity prevention in England: a qualitative study.* BMC Public Health, 2015. **15**: p. 1224.

5. St George, S.M., Messiah, S.E., Sardinas, K.M., et al., *Familias Unidas for Health and Wellness: Adapting an Evidence-Based Substance Use and Sexual Risk Behavior Intervention for Obesity Prevention in Hispanic Adolescents.* J Prim Prev, 2018. **39**(6): p. 529-553.

6. Jurkowski, J.M., Green Mills, L.L., Lawson, H.A., et al., *Engaging low-income parents in childhood obesity prevention from start to finish: a case study.* J Community Health, 2013. **38**(1): p. 1-11.

7. Waters, E., Gibbs, L., Tadic, M., et al., *Cluster randomised trial of a school-community child health promotion and obesity prevention intervention: findings from the evaluation of fun 'n healthy in Moreland!* BMC Public Health, 2017. **18**(1): p. 92.
