# Supplementary material for: Valuing Australian parent preferences for community-based nutrition and physical activity initiatives: a discrete choice experiment
Source: Health Promot Int. 2026 Mar 9;41(2):daag033. doi: 10.1093/heapro/daag033 (PMC13017149; doi:10.1093/heapro/daag033)
Supplement: daag033_Supplementary_Data [file daag033_supplementary_data.zip › Supplementary file 4 Attitudinal variables.docx]

**Supplementary file 4 Attitudinal variables**

| **How worried are you about your child experiencing overweight and obesity?**  Not worried at all  A little bit worried  Moderately worried  Very worried  Extremely worried | 139 (31.7%)  151 (34.4%)  88 (20.1%)  39 (8.9%)  21 (4.8%) |
| --- | --- |
| **How worried should parents be about their child experiencing overweight and obesity?**  Not worried at all  A little bit worried  Moderately worried  Very worried  Extremely worried | 15 (3.4%)  98 (22.4%)  187 (42.7%)  89 (20.3%)  49 (11.2%) |
| **Childhood obesity is a serious public health issue.**  Strongly disagree  Somewhat disagree  Neither agree nor disagree  Somewhat agree  Strongly agree | 7 (1.6%)  15 (3.4%)  62 (14.2%)  196 (44.8%)  158 (36.1%) |
| **Climate change is a serious concern.**  Strongly disagree  Somewhat disagree  Neither agree nor disagree  Somewhat agree  Strongly agree | 20 (4.6%)  37 (8.5%)  61 (13.9%)  152 (34.7%)  168 (38.4%) |
| **How worried should parents be about climate change impacting on their child’s health?**  Not worried at all  A little bit worried  Moderately worried  Very worried  Extremely worried | 48 (11.0%)  73 (16.7%)  127 (29.00%)  102 (23.3%)  88 (20.1%) |
| **I am concerned about the impact of climate change on future generations.**  Strongly disagree  Somewhat disagree  Neither agree nor disagree  Somewhat agree  Strongly agree | 25 (5.71%)  19 (4.34%)  64 (14.61%)  163 (37.21%)  167 (38.13%) |
| **How confident do you feel to support your child in participating in a healthy lifestyle initiative?**  Not at all confident  Slightly confident  Somewhat confident  Very confident  Extremely confident | 6 (1.4%)  31 (7.1%)  121 (27.6%)  178 (40.6%)  102 (23.3%) |
