# Supplementary material for: Valuing Australian parent preferences for community-based nutrition and physical activity initiatives: a discrete choice experiment
Source: Health Promot Int. 2026 Mar 9;41(2):daag033. doi: 10.1093/heapro/daag033 (PMC13017149; doi:10.1093/heapro/daag033)
Supplement: daag033_Supplementary_Data [file daag033_supplementary_data.zip › Supplementary file 5 DIRECT checklist.docx]

**DIRECT checklist**

Checklist for reporting discrete choice experiments in health ^[236]^

| Section  item |  | Page and  Paragraph no |
| --- | --- | --- |
| Purpose and rationale | | |
| 1 | Describe the real-world context and decision-maker that the hypothetical choice context seeks to replicate or inform | Pages 5-6  Para 2-1 |
| 2 | Provide a rationale for using a DCE to answer the research question | Page 7  Para 2 |
| Attributes and levels | | |
| 3 | Describe how attributes and levels were derived | Page 8  Para 2-3 |
| 4 | Provide the final list of attributes and levels | Page 9  Para 3 |
| Experimental design | | |
| 5 | Report the number of alternatives per choice set and whether they were labelled or unlabelled | Page 10 Para 2 |
| 6 | Describe response options | Page 10  Para 2 |
| 7 | Describe the type of experimental design | Page 12  Para 1 |
| 8 | Describe which effects are identified in the design | Page 13  Para 2 |
| 9 | Describe the number of choice sets, blocks and choice sets per block | Page 10  Para 2 |
| 10 | Indicate how the experimental design was obtained | Page 13  Para 1 |
| Survey design | | |
| 11 | Provide a sample choice set and the instructions and background information given to respondents | Page 11  Para 1 |
| 12 | Report any randomisation | Page 6  Para 3 |
| 13 | Describe what was checked in piloting | Page 9 &12  Para 2 |
| 14 | Report whether information from the pilot was used to update the experimental design | Page 12  Para 2 |
| Sample and data collection | | |
| 15 | Report respondent inclusion/exclusion criteria | Pages 10-11  Para 1-4 |
| 16 | Describe how data were collected | Page 11  Para 2 |
| 17 | Report the response rate or cooperation rate, if possible | Page 14  Para 2 |
| 18 | Report the final sample size and how the sample size was determined | Pages12Para 2 |
| 19 | Describe respondent characteristics and representativeness of target population | Pages 11-12  Para 2-1 |
| Economic analysis | | |
| 20 | Indicate coding of data | Page 10  Para 1 |
| 21 | Report whether any respondents were removed and why | Page 14  Para 2 |
| 22 | Provide the rationale for model choice and assumptions | Page 13  Para 3 |
| 23 | Report model specification | Page 13  Para 2 |
| Reporting of results | | |
| 24 | Report the model performance, goodness of fit (if comparing models) | Not reported |
| 25 | Describe methods used for analysis of model results | Page 13  Para 1 |
| 26 | Report measures of precision for the output(s) of interest | Page 15  Para 1 |
