# Supplementary figures and images for: Valuing Australian parent preferences for community-based nutrition and physical activity initiatives: a discrete choice experiment
Source: Health Promot Int. 2026 Mar 9;41(2):daag033. doi: 10.1093/heapro/daag033 (PMC13017149; doi:10.1093/heapro/daag033)

Supplementary File 2 Choice task example


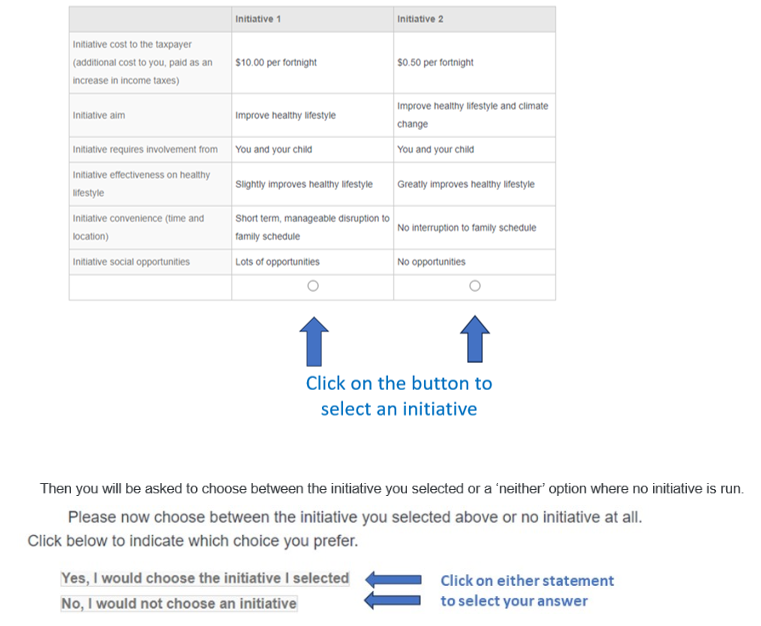

Supplement: daag033_Supplementary_Data [file daag033_supplementary_data.zip › Supplementary File 2 Choice task example.docx]
